# The neonatal Fc receptor (FcRn): Guardian or Trojan Horse in viral infection?

**DOI:** 10.1371/journal.ppat.1013285

**Published:** 2025-07-07

**Authors:** Lei Na, Yang Zheng, Jian-Bo Yang, Hao-Lin Bao, Yan-Dong Tang

**Affiliations:** 1 College of Animal Husbandry and Veterinary Medicine, Jiangsu Vocational College of Agriculture and Forestry, Jurong, China; 2 State Key Laboratory for Animal Disease Control and Prevention, Harbin Veterinary Research Institute of Chinese Academy of Agricultural Sciences, Harbin, China; UW-Madison: University of Wisconsin Madison, UNITED STATES OF AMERICA

## Abstract

The neonatal Fc receptor (FcRn), well-known for mediating the transfer of maternal immunoglobulin G (IgG) to neonates, plays a critical role in neonatal antimicrobial defenses. Furthermore, FcRn regulates IgG and albumin homeostasis via pH-dependent recycling pathways, thereby prolonging their plasma half-life in circulation. FcRn also enables bidirectional transcytosis of antibodies across cellular barriers, a function essential for maintaining humoral immunity. However, recent studies have demonstrated that some viruses exploit FcRn to facilitate viral infection, underscoring its dual role in the viral lifecycle. This review aims to comprehensively discuss the dual functions of FcRn during viral infections, with particular focus on how diverse viruses utilize FcRn to promote infection. Here, we summarize the molecular mechanisms underlying FcRn-mediated proviral processes, including viral uncoating, transcytosis, and antibody-dependent enhancement (ADE) of infection. Finally, we propose potential therapeutic strategies targeting FcRn to inhibit viral replication.

## Introduction

Historically, viral pathogens have dominated the landscape of human infectious diseases, serving as the primary causative agents for the majority of emerging or newly recognized epidemics [1]. The 21st century alone has witnessed recurrent global health crises driven by viral outbreaks, including the 2003 SARS-CoV-1 epidemic, the 2009 H1N1 influenza pandemic, the 2012 MERS-CoV emergence, the 2013–2016 Ebola epidemic in West Africa, the 2015 Zika virus outbreak, and the ongoing COVID-19 pandemic caused by SARS-CoV-2 [1,2]. Furthermore, outbreaks of viruses, such as African swine fever virus and avian influenza virus, in animals can also have substantial impacts on our society [3–5]. These outbreaks resulted in widespread morbidity and mortality, underscoring the persistent threat posed by viral pathogens. It is fortunate that hosts have developed multilayered defense mechanisms against viruses. For example, humoral immunity, led by immunoglobulin G (IgG), plays a pivotal role in antiviral protection. IgG, accounting for approximately 80% of circulating immunoglobulins, acts as the primary mediator of antibody-dependent antiviral responses [6]. Structurally, IgG adopts a Y-shaped conformation formed by two disulfide-linked γ heavy chains and two light chains (κ or λ), which collectively generate antigen-binding fragments (Fab) and a crystallizable fragment (Fc). This architecture enables IgG to mediate diverse effector functions, including antigen neutralization, complement activation, and interactions with Fc receptors [7]. The human IgG family comprises four distinct subclasses-IgG1, IgG2, IgG3, and IgG4-defined by unique γ-heavy chain sequences and ranked in descending order of their baseline plasma levels [8]. Key structural distinctions between subclasses arise from variations in hinge region length and the quantity/position of inter-heavy chain disulfide bonds, which critically influence their functional diversity [6]. Diverse antibody isotypes collectively form the cornerstone of humoral immunity against microbial invasion, orchestrating pathogen neutralization and clearance through multiple effector mechanisms [6]. Recent advances have further delineated IgG’s multifaceted roles in antiviral defense, such as neutralizing virions by blocking receptor engagement, orchestrating antibody-dependent cellular phagocytosis and cytotoxicity, and modulating inflammatory cascades to influence clinical outcomes [7].

Despite the key role of IgG in adaptive immunity, newborns lack fully functional adaptive immune systems. Passive immune protection mediated by IgG during this developmental period is facilitated by the neonatal Fc receptor (FcRn). This phenomenon initially discovered by Brambell and colleagues through landmark studies demonstrating maternal IgG transport to offspring across the neonatal rodent intestinal epithelium [9,10]. In fact, FcRn transfers maternal IgG to offspring either via transplacental transport or through uptake of IgG from milk in the proximal small intestine. Over the past decade, research has significantly advanced our understanding of FcRn biology. While rodents rely on intestinal FcRn for postnatal IgG acquisition, humans utilize placental FcRn to transfer maternal IgG to the fetus during gestation, ensuring prenatal immune protection [11]. Beyond its neonatal function, FcRn is now recognized as a lifelong regulator of IgG and albumin homeostasis, extending their serum half-lives through pH-dependent cellular recycling mechanisms [12,13]. Although many important prior comprehensive reviews have detailed FcRn’s contributions to humoral immunity and albumin homeostasis [14–16], this review will focus on the emerging role of viral pathogens in exploiting FcRn to promote viral replication. Herein, we will summarize its dual roles in viral infections, while emphasizing translational opportunities for targeting FcRn in vaccine design and antiviral therapeutic development.

## Structural characteristics

FcRn is a cell-surface glycoprotein belonging to the type I membrane protein family, it is encoded by the *FCGRT* gene and structurally organized into three extracellular α-helical domains (α1, α2, α3), a single-pass transmembrane segment, and an intracellular C-terminal region [17]. The α1 and α2 domains generate a constricted cleft that, despite lacking peptide-presenting capacity, cooperatively assembles with the membrane-proximal α3 domain and β2-microglobulin (β2m) to establish the receptor’s ligand-binding architecture [17]. This heterodimer is essential for its stability and ligand binding [18,19]. Although evolutionarily related to MHC class I molecules, FcRn diverges from this family by lacking a peptide-binding groove while preserving its unique pH-dependent binding specificity for IgG and albumin [20]. Mechanistically, IgG engages FcRn at the CH2-CH3 domain interface exclusively under acidic conditions (pH < 6.5), with dissociation occurring at neutral physiological pH [21]. This pH-sensitive binding modality constitutes the biochemical cornerstone of FcRn’s physiological functions [14–16]. The broad tissue expression profile of FcRn reflects its multifaceted physiological roles. FcRn is widespread in endothelial cells, epithelial barriers (e.g., placenta, lungs, intestines), and immune cells (including dendritic cells and macrophages), underscoring its systemic physiological significance [14,22]. Functionally, FcRn orchestrates two pivotal processes: (1) pH-dependent recycling of IgG and albumin, which prevents lysosomal degradation and prolongs their circulatory persistence, and (2) bidirectional IgG transcytosis across cellular barriers, facilitating immune surveillance and pathogen neutralization [14–16]. Notably, polarized epithelial cells employ FcRn as the exclusive mediator of bidirectional IgG transport, contrasting with the unidirectional translocation of polymeric IgA/IgM mediated by the polymeric immunoglobulin receptor [23]. This mechanism underpins critical immunological defenses at epithelial interfaces, where IgG exerts essential protective roles. Leveraging its unique structural and functional properties, FcRn serves as a pivotal mediator of antiviral immune defense mechanisms. Paradoxically, viral pathogens have evolved sophisticatedly to subvert this pathway, hijacking the FcRn-mediated transport system to potentiate viral infection. The molecular intricacies of these immune evasion strategies, as well as their implications for viral pathogenesis, will be discussed in following sections.

## Guardian role of FcRn in viral infection

### IgG transfer from mother to offsprings

**Fig 1:**
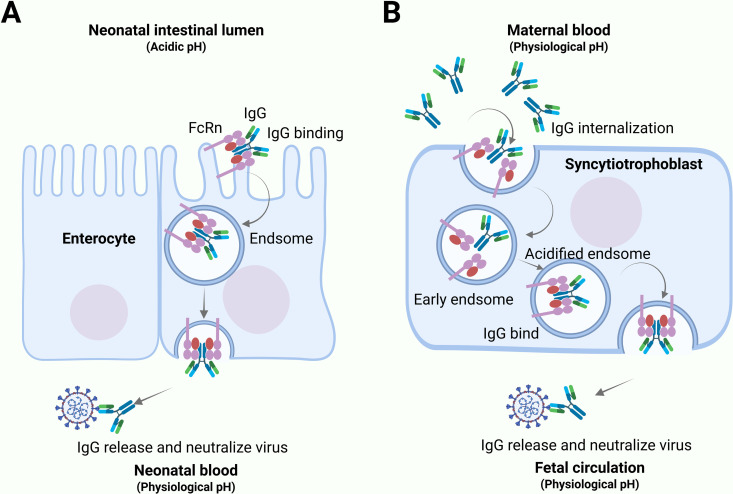
IgG transfer from mother to offsprings. (**A**) In neonatal rodents, maternal IgG is processed in the stomach and enters the duodenum (pH ~ 6.0). Here, IgG binds to FcRn on duodenal epithelial cells, which transports it across the cell via transcytosis and releases it into the basolateral space at physiological pH (7.4). (**B**) In humans, placental cells internalize maternal IgG. Acidification of endosomes (pH ~ 6.0) strengthens IgG-FcRn binding. Vesicles traffic to the fetal side, where neutral pH (7.4) triggers IgG release. The schematic, inspired by reference [14], was redrawn using BioRender.com.

When newborns encounter viral infections, they rely on passive immune protection mediated by maternal IgG. Maternal IgG is transferred to the offspring via FcRn-mediated transport mainly at two primary sites: the placenta in humans and the proximal small intestine in rodents. In neonatal rodents, maternal IgG undergoes gastric processing before entering the duodenum with mildly acidic gastric contents (pH ~ 6.0). At this site, IgG binds to apically localized FcRn on duodenal epithelial cells. FcRn then mediates transcytosis of the IgG, ultimately releasing it into the basolateral extracellular space at physiological pH (7.4) (Fig 1A) [15,16]. In contrast, human syncytiotrophoblasts internalize maternal IgG-containing fluid via endocytosis. The progressive acidification of these endosomes (pH ~ 6.0) induces high-affinity IgG-FcRn binding within this compartment. Following vesicular trafficking to the fetal-facing membrane, physiological pH (7.4) triggers IgG dissociation. The FcRn receptor subsequently recycles to the maternal membrane for repeated transcytosis cycles, a mechanism conserved across polarized transport systems (Fig 1B) [14–16]. While placental FcRn mediates transplacental IgG transfer during gestation in humans, FcRn in the fetal and neonatal intestinal epithelium also facilitates IgG uptake from swallowed amniotic fluid *in utero* and from breast milk, respectively [24,25]. Regardless of the specific pathway by which IgG is transferred from mother to offspring, FcRn plays a key protective role in combating viral infections in the offspring.

### FcRn prolongs IgG half-life

**Fig 2:**
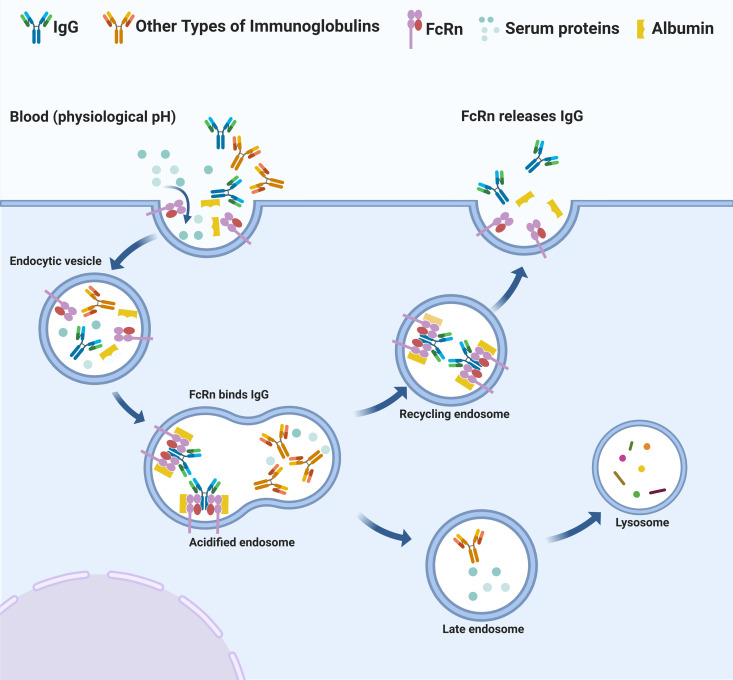
FcRn prolongs IgG and albumin half-life. FcRn binds IgG or albumin in acidic endosomes and transports them to the cell surface, where neutral pH (7.4) triggers release into circulation. This pH-dependent recycling extends half-life of IgG and albumin from days to weeks, maintaining protective serum levels. The schematic, inspired by reference [14], was redrawn using BioRender.com.

An effective antiviral strategy should combine potent antiviral activity with prolonged plasma half-life to maintain therapeutic coverage throughout infection. For IgG, its long half-life is achieved through FcRn, which extends IgG antibody persistence via a pH-dependent recycling mechanism. FcRn extends IgG and albumin persistence by protecting them from lysosomal degradation through a pH-dependent recycling mechanism. In neonatal endothelial and hematopoietic cells, FcRn binds IgG and albumin within acidic endosomes and subsequently transports it back to the cell surface. The antibody is released into circulation upon exposure to physiological pH (7.4), effectively increasing half-life of IgG and albumin from days to weeks and sustaining protective serum concentrations (Fig 2) [14–16]. This prolonged antibody presence, particularly the sustained levels of pathogen-specific neutralizing antibodies, is critical for ensuring antiviral protection over an extended period following viral infection.

### FcRn’s role in antigen presentation

Emerging studies have highlighted significant FcRn expression in hematopoietic cells, particularly within myeloid lineage cells such as monocytes, tissue-resident macrophages, dendritic cells (DCs), and neutrophils [16,26]. This specific distribution allows FcRn-positive cells to differentially handle monomeric IgG versus multimeric immune complexes (ICs). Binding to monomeric IgG promotes antibody stabilization and prolongs serum persistence, whereas engagement with ICs initiates distinct trafficking pathways to specialized antigen-processing compartments. Subsequent degradation of these complexes releases antigens for enhanced presentation via MHC class I and II, while also amplifying the production of proinflammatory cytokines. Collectively, these processes collectively shape adaptive immune responses [27–29].

### Mediated intracellular viral neutralization

IgG has classically been viewed to neutralize virions through extracellular mechanisms, such as blocking viral attachment to its receptors. However, emerging evidence now reveals an intracellular antiviral paradigm [30–32]. A striking example is the influenza hemagglutinin-specific monoclonal antibody Y8-10C2 (Y8), which exhibits pH-dependent neutralizing activity [30]. In polarized Madin-Darby canine kidney monolayers expressing rat FcRn, basolateral application of Y8 significantly suppressed viral replication following apical influenza infection. This neutralization strictly required FcRn expression and its IgG transcytosis function. Given the shared pH dependence of FcRn-IgG binding (pH 6.0–6.5) and Y8’s optimal antiviral activity under acidic conditions, these findings strongly suggest an intracellular neutralization mechanism. Confocal microscopy revealed tripartite colocalization of influenza virions, Y8, and FcRn within endosomal compartments [30]. This spatial convergence implies two potential inhibitory mechanisms: (1) interference with pH-dependent viral envelope fusion to endosomal membranes during primary uncoating, and (2) blockade of nuclear translocation of viral nucleoprotein antigens. The therapeutic relevance of this pathway was demonstrated *in vivo*: prophylactic Y8 administration protected wild-type mice from lethal influenza challenge, markedly reducing pulmonary viral titers and attenuating virus-induced lung pathology. However, FcRn-knockout mice showed complete loss of Y8-mediated protection, confirming FcRn’s indispensable role in this intracellular defense system. These findings establish a non-canonical viral neutralization axis wherein FcRn transports pathogen-specific IgG into endocytic pathways, enabling interception of virions during intracellular trafficking. This mechanism expands the functional repertoire of humoral immunity beyond extracellular neutralization.

## Trojan FcRn in viral infection

### FcRn as a multifunctional viral entry receptor

**Fig 3:**
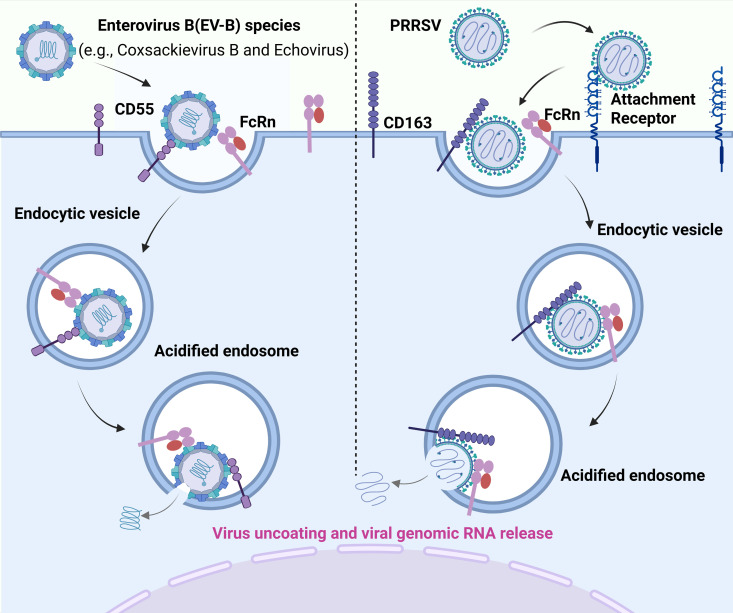
FcRn as a multifunctional viral entry receptor. (**A**) Enterovirus B (EV-B) members (e.g., Coxsackievirus B, Echovirus) attach to cells via CD55 or integrins, while FcRn acts as their exclusive uncoating receptor to enable RNA release. (**B**) For PRRSV entry into porcine alveolar macrophages, the virus first binds to potential attachment receptors, including heparan sulfate, vimentin, sialoadhesin, MYH9, DC-SIGN, or CD151. The virus is then internalized, and FcRn and CD163 mediate endosomal uncoating. Acidic endosomal conditions allow FcRn to destabilize the virion and facilitate the release of viral genomic RNA into the host cytoplasm. The figure was created with BioRender.com.

Enveloped viruses typically initiate host cell invasion through either direct membrane fusion at the plasma membrane or endocytic trafficking to specialized compartments where pH-dependent conformational changes trigger membrane fusion [33,34]. Non-enveloped viruses employ a sophisticated biphasic entry strategy: initial cell surface attachment followed by capsid destabilization and genome release [33,34]. While some viruses utilize distinct receptors for these sequential steps, others have evolved elegant single-receptor systems capable of mediating both attachment and uncoating. Within Enterovirus B (EV-B) species (e.g., Coxsackievirus B and Echovirus), cellular attachment primarily occurs through CD55 or integrins (α2β1/αVβ6/αVβ3), with FcRn serving as the dedicated uncoating receptor [35,36]. Notably, Echovirus 18 demonstrates an evolutionary adaptation where FcRn uniquely fulfills both attachment and uncoating functions [37]. FcRn-mediated uncoating occurs post-endocytosis, where the acidic endosomal environment (pH 6.0–6.5) induces FcRn binding to conserved structural motifs of viral capsid protein VP1. Structural studies using cryo-electron microscopy (cryo-EM) demonstrate that FcRn binding induces conformational changes in the VP1 canyon, destabilizing the capsid and facilitating RNA release (Fig 3) [35]. This FcRn-dependent uncoating paradigm extends from enteroviruses to enveloped viruses. The porcine reproductive and respiratory syndrome virus (PRRSV), a destructive arterivirus (Family: *Arteriviridae*), causes significant economic losses in global swine production [38–40]. In a recent study, PRRSV entry target cells relies on FcRn for endosomal uncoating within alveolar macrophages [41]. While FcRn also functions as an essential entry receptor for arteriviruses, including simian hemorrhagic fever virus (SHFV) and equine arteritis virus (EAV), whether FcRn serves similar uncoating functions in these viruses remains to be confirmed [42]. PRRSV exhibits remarkable receptor plasticity during initial infection, engaging multiple attachment factors including heparan sulfate, vimentin, CD169 (sialoadhesin), MYH9, DC-SIGN (CD209), and CD151 for cell surface docking [43]. Following clathrin-mediated internalization, FcRn orchestrates viral uncoating through direct interactions with the viral nucleocapsid (N) protein and membrane (M) glycoprotein in acidified endosomes (Fig 3) [41]. Notably, prior to FcRn identification, the scavenger receptor CD163 had been established as another essential PRRSV uncoating factor [43,44]. Furthermore, CD163 abundance in its target cells is a pivotal switch for PRRSV infection [45]. However, reciprocal overexpression experiments reveal non-redundant functions: CD163 overexpression fails to compensate for FcRn deficiency, and vice versa. This strongly suggesting CD163 and FcRn are coordinated receptors rather than functional overlap during PRRSV uncoating. Collectively, these findings posit that CD163 and FcRn may orchestrate PRRSV uncoating through sequential engagement with distinct viral structural components, potentially via spatiotemporal cooperation. Nevertheless, the exact mechanistic hierarchy governing viral co-option of these receptors remains poorly characterized and requires further investigation.

Recent studies have shown that Astroviruses, particularly human astrovirus (HAstV), also use FcRn as a primary receptor for cellular entry [46,47]. Studies using CRISPR-Cas9 knockout models demonstrate that deletion of FcRn subunits (FCGRT or β2M) renders cells resistant to HAstV infection. Conversely, introducing human FcRn into non-susceptible cells (e.g., HEK293) confers viral permissibility, confirming its necessity and sufficiency for infection [46]. FcRn directly binds to the HAstV spike protein (VP25) with high affinity, as shown by surface plasmon resonance assays [46]. However, astrovirus spike proteins engage FcRn at a distinct site, independent of IgG or albumin binding, enabling simultaneous interactions with multiple host receptors, such as dipeptidyl-peptidase IV (DPP4) [46]. Overall, they demonstrated FcRn functions as a cellular receptor for HAstV infection, and DPP4 as an entry co-factor. Additional research is required to establish whether FcRn also serves as both a binding and uncoating receptor for viral entry.

### FcRn-mediated viral transcytosis

**Fig 4:**
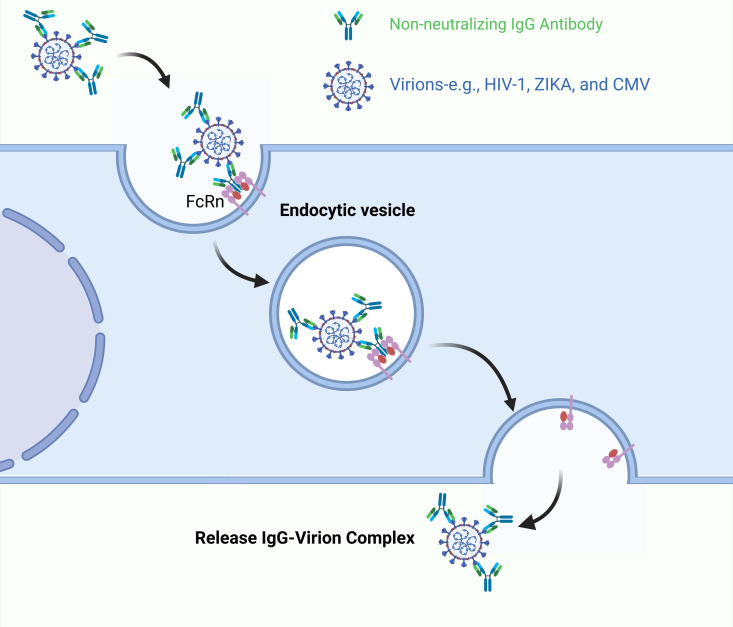
FcRn-mediated viral transcytosis. Cross-reactive or non-neutralizing antibodies recognizing viruses such as HIV-1, CMV, or ZIKV form virion-antibody complexes that hijack FcRn. This FcRn-dependent pathway mediates transcellular infection across epithelial cells or syncytiotrophoblasts. The figure was created with BioRender.com.

Emerging evidence reveals that multiple pathogenic viruses, including human immunodeficiency virus type 1 (HIV-1), Zika virus (ZIKV), and human cytomegalovirus (CMV), have evolved convergent strategies to exploit FcRn to traverse cellular barriers and establish systemic infection (Fig 4) [48–51]. For instance, non-neutralizing IgG antibodies in mucosal secretions bind HIV-1, forming IgG-Virion complexes that engage FcRn on genital epithelial cells. Under acidic luminal conditions (pH ~ 6.0), FcRn facilitates the transcytosis of these complexes across the epithelial barrier, ultimately depositing infectious virions at the basolateral surface for subsequent infection [48]. The placental transmission of ZIKV demonstrates an intriguing example of cross-reactive immune enhancement. Maternal IgG antibodies generated during prior dengue virus (DENV) exposure paradoxically enhance ZIKV infectivity through antibody-dependent enhancement (ADE) mechanisms [49]. These cross-reactive, non-neutralizing antibodies form complexes with ZIKV particles that effectively hijack FcRn transport pathways in syncytiotrophoblasts. This receptor-mediated exploitation allows vertical viral transmission despite placental barrier defenses, highlighting a dangerous immunological interplay between flavivirus exposures [49]. CMV pathogenesis reveals additional complexity in FcRn utilization. Syncytiotrophoblast apical microvilli demonstrate pH-dependent binding of IgG-CMV complexes through FcRn receptors, with transcytosis completion occurring within 1 h as demonstrated by basal compartment detection [51]. Experimental inhibition of FcRn through competitive Fc fragment saturation or protein A treatment completely blocks this transport process. The fate of transcytosed complexes depends on antibody quality: virions associated with low-titer neutralizing antibodies maintain infectivity post-transport, while those bound by high-titer neutralizing antibodies undergo capture by villus core macrophages. This conserved mechanism of FcRn-mediated transcytosis across divergent viral species underscores its evolutionary significance as a pathogenic strategy. The shared capacity to exploit IgG–FcRn interactions for barrier penetration suggests this pathway may represent a critical vulnerability for vertical transmission of CMV and potentially other congenital viral infections. These findings emphasize the dual role of humoral immunity in both protection and pathogenic facilitation, necessitating careful consideration in vaccine development and therapeutic antibody design.

## Concluding remarks and future perspectives

### Rational FcRn-based vaccine design

Vaccination remains a cornerstone strategy for controlling viral infectious diseases [52,53]. However, most vaccinations rely on intramuscular administration, which may exhibit limited efficacy against respiratory or enteric pathogens due to inadequate induction of systemic mucosal immunity. Recent breakthroughs highlight FcRn as a central regulator of mucosal immunity, leveraging its unique capacity to mediate transcytosis of IgG-antigen complexes across respiratory epithelial barriers. By utilizing this pathway, vaccines based on IgG Fc-fused antigens can orchestrate multilayered protection against diverse respiratory and enteric viruses, including influenza virus, SARS-CoV-2, porcine epidemic diarrhea virus (PEDV), and pseudorabies virus (PRV) [54–57]. Mimicking natural IgG trafficking, FcRn efficiently delivers IgG Fc-fused antigens to mucosal inductive sites, overcoming the limitations of traditional intramuscular vaccines that inadequately activate respiratory immunity. The platform’s ability to leverage physiological IgG transport pathways offers distinct advantages over conventional vaccination approaches in preventing viral infection and transmission.

### Viruses-resistant breeding via precision gene editing

Precision gene editing has become a transformative tool for enhancing disease resistance in livestock. By identifying key functional domains in host-pathogen interactions, this approach enables genomic modifications to block viral replication while maintaining host protein functionality. This strategy is particularly vital for combating PRRSV, a major threat to swine health. CRISPR-Cas9 technology has already achieved breakthroughs, such as developing PRRSV-resistant pigs through editing of CD163, a critical receptor for PRRSV [58–60]. FcRn, with its dual role in IgG regulation and potential involvement in PRRSV infection, it may use as a promising target for generating next-generation PRRSV-resistant pigs. To advance these efforts, more studies into viral mechanisms and host entry pathways are essential. Integrating structural biology, immunogenetics, and protein modeling will enable the design of livestock with broad-spectrum disease resistance.

### Antiviral therapy

For viral pathogens that exploit FcRn to facilitate replication and dissemination, innovative antiviral approaches can be designed through antibody engineering. A dual-target strategy involves the development of modified IgG Fc fragments engineered to either competitively occupy viral attachment sites or sterically hinder IgG-virion complex interactions with FcRn. While this interference mechanism may inhibit viral replication, its therapeutic implementation faces a constraint: potential attenuation of vaccine-induced protective antibody titers through FcRn competition. An alternative approach focuses on engineering neutralizing antibodies with structurally optimized Fab domains. These modified antigen-binding regions maintain high-affinity viral antigen recognition while undergoing precision mutagenesis to eliminate FcRn-binding capacity in the Fc region. This molecular redesign achieves dual therapeutic objectives by blocking FcRn-mediated viral entry/transcytosis mechanisms while preserving humoral immune functions.

In recent years, FcRn has emerged as a transformative therapeutic target for autoimmune diseases owing to its pivotal role in regulating IgG homeostasis [61]. Current drug development initiatives are focused on inhibiting FcRn-IgG interactions to accelerate the clearance of pathogenic antibodies. Several candidates are currently undergoing clinical trials to evaluate their efficacy in treating myasthenia gravis and other autoimmune conditions [62]. These inhibitors also exhibit potential significance in combating viral infections that utilize FcRn-mediated pathways. For instance, nipocalimab effectively blocks HAstV1 infection, and orilanolimab shows promise as a potent arterivirus-blocking agent [42,46]. Overall, FcRn may be used as a key target for antiviral therapy in near future.
